# Sticky plants and plant-based glues: potential for pest control

**DOI:** 10.3389/fpls.2025.1612368

**Published:** 2025-07-08

**Authors:** Thijs V. Bierman, Young H. Choi, T. Martijn Bezemer

**Affiliations:** ^1^ Above-Belowground Interactions Group, Institute of Biology, Sylviusweg, Leiden, Netherlands; ^2^ Natural Products Laboratory, Institute of Biology Leiden, Sylviusweg, Leiden, Netherlands

**Keywords:** trichomes, bio-based, arthropod trapping, biological control, agriculture

## Abstract

Many vascular plants produce adhesive substances that may trap arthropods for their own protection, nutrition, and to engage in mutualistic relationships with predatory arthropods. While the role of stickiness in plant defense is well established, our understanding of the mechanisms and factors that determine the successful capture of arthropods by sticky plants and how we can utilize this knowledge to increase the sustainability of our agricultural practices is still limited. We review the literature on arthropod-trapping sticky plants and plant-based adhesive use in agriculture. There are many factors involved in the successful capture of arthropods by sticky plants, including: plant morphology, glue chemistry, the use of visual cues and volatiles to affect arthropod behavior, environmental factors, and adaptations of arthropods in their behavior, morphology, and chemistry to avoid being captured. Considering agricultural potential, using sticky crops as trap plants and ameliorating crops with sticky features could be useful for crop protection, but practical application is scarce. The same is true for the use of sticky plant specialist arthropod predators. Furthermore, plant-based adhesives are becoming more popular in agriculture for example, as glues for sticky traps, as sprayable adhesives for physical plant protection, and as carriers of botanicals and pesticides. So far, these adhesives see only small-scale use and are often less effective in the field than in the laboratory. Before plant stickiness and plant-based glues can be fully utilized for crop protection, several technological and resource related challenges must also first be overcome.

## 1 Introduction

### 1.1 The world of sticky plants

For millions of years, plants have existed in various environments and coexisted with arthropods and other organisms. To survive and thrive, plants utilize a wide array of strategies, including the use of sticky substances to repel, hinder and trap arthropods (Darwin, 1875). One of the most common plant structures from which adhesive mucilage or resin may be secreted are glandular trichomes: epidermal plant hairs with specialized gland cells that contain and/or release various metabolites (Wagner, 1991). Plants with such trichomes are often described as “sticky”, “adhesive”, “clammy”, “glandular”, “glutinous”, “gummy”, “resinous”, “viscid”, “tarry”, “tacky”, or “mucilaginous”; terminology that may also be found in their common and scientific names (Wheeler and Krimmel, 2015). In this review, we consider sticky plants to be those plants that secrete adhesive mucilage or other adhesive fluids, either via glandular trichomes or other gland types, that can trap other organisms.

**Figure 1:**
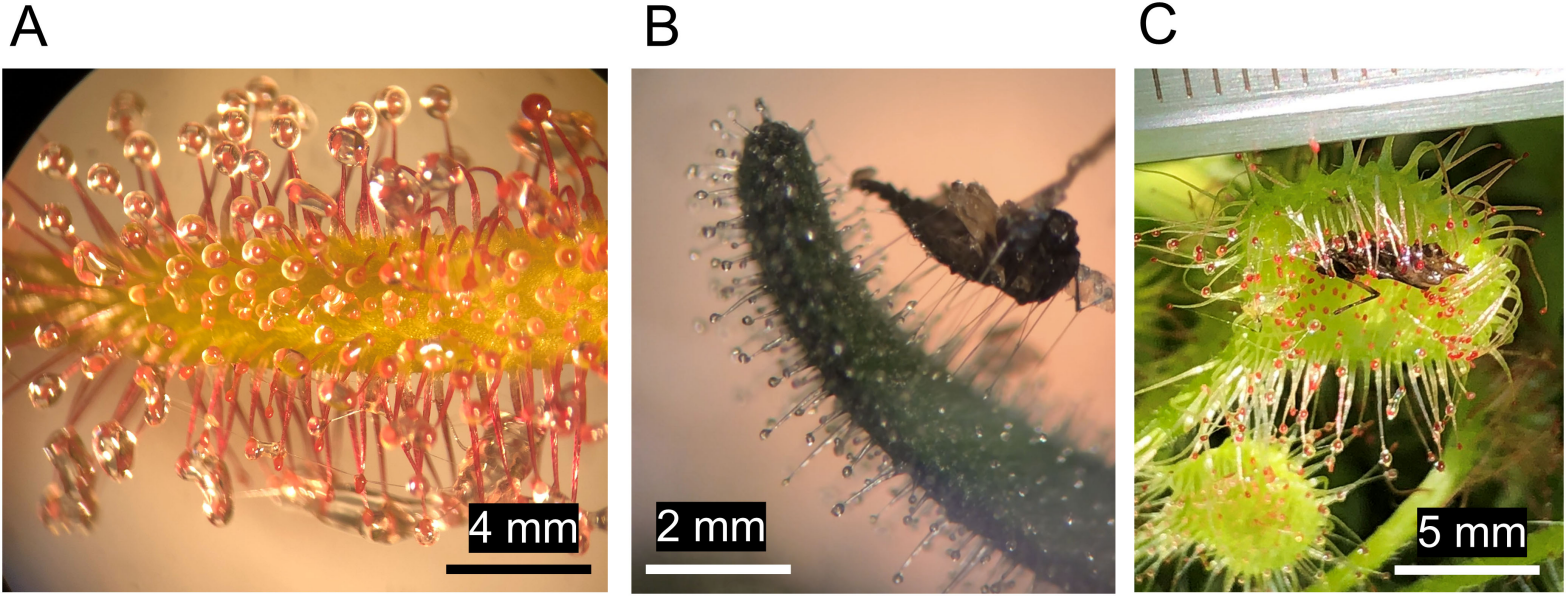
The glandular trichomes of the carnivorous plant *Drosera capensis* are covered in polysaccharide-rich mucilage, their shimmer is thought to attract flies and other arthropods. Trapped arthropods are digested using enzymes present in the mucilage. In this way, the plant obtains nitrogen and other nutrients **(A)**. Small arthropods frequently become stuck in the secretions of glandular trichomes. Here, a carcass of a fungus gnat (*Diptera*) is being pulled from the glandular trichomes on the sepal of a sticky *Petunia* cultivar. Polysaccharide-based glues, like those of *Petunia* rely on capillary forces to adhere insects and can stretch great lengths before breaking **(B)**. The trichomes of some sticky plants can bend to facilitate better prey retention and digestion. Here, a *Drosera rotundifolia* leaf with its trichomes bent around a captured insect **(C)**.

Plants from over 110 genera in 49 families have already been identified to possess arthropod-trapping abilities, and most often this is due to their stickiness (LoPresti et al., 2015). Famous examples of arthropod-trapping sticky plants include carnivorous and protocarnivorous species, for example: *Pinguicula* spp., *Byblis* spp., *Roridula* spp., and Darwin’s beloved *Drosera* spp (Chase et al., 2009; Adlassnig et al., 2010). These plants possess specialized, often elongated glandular trichomes that secrete adhesive mucilage or resin-like substances that allow the plants to immobilize their prey (**Figures 1A-C**) (Li et al., 2023). Captured arthropods drown or starve to death and are then decomposed using enzymes (e.g., proteases and phosphatases) or by other organisms like microbes, fungi, or carrion feeding arthropods (Peroutka et al., 2008). In this way, these arthropod-trapping plants can obtain essential nutrients, especially nitrogen, phosphorus, and other trace elements (Ellison, 2006; Adlassnig et al., 2012), which contribute to healthy plant growth and increased fitness (Givnish et al., 2018; Klink et al., 2019). There are also many (presumably) non-carnivorous species of sticky plants, including herbs such as *Datura wrightii*, *Madia elegans, Geranium* spp., crop species such as cotton (*Gossypium* spp.), tomato (*Solanum lycopersicum*) and tobacco (*Nicotiana tabacum*) (LoPresti et al., 2015; Nelson et al., 2019a), and woody plants such as *Rhododendron macrosepalum* (Sugiura and Yamazaki, 2006) and horse-chestnut (*Aesculus hippocastanum*) (Voigt et al., 2020). The density of mucilage producing trichomes or general stickiness of these plants is often the greatest around the reproductive organs such as buds, flowers, and fruits (LoPresti et al., 2018b; Chautá et al., 2022). Some trees of the genus *Pisonia* are even known to occasionally trap birds in the mucilage surrounding their seedpods (Burger, 2005).

### 1.2 Review aim and methods

There are many studies, books (e.g., Adlassnig et al., 2010) and reviews (e.g., Wheeler and Krimmel, 2015; Mithöfer, 2022; Luna-Samano et al., 2024), that describe the ecology of sticky plants and their interactions with arthropods. This review aims to build on this knowledge to seek a deeper understanding of what determines successful arthropod capture in sticky plants, and in what ways we can utilize these plants, their associated predatory arthropods, and other plant-derived sticky materials for crop protection. First, we summarize the mechanisms and factors involved in the successful trapping of arthropods by naturally sticky plants. Then, we will highlight some of the most recent developments on how sticky plants, natural adhesiveness, the predators living on sticky plants, and plant-derived glues can be and are already applied in agriculture for arthropod pest control, with an emphasis on integration with integrated pest management.

Initially, a systematic search was done for literature on sticky plants on May 1^st^ 2024 in Web of Science, using a specific search term: “(adhesi* OR stic* OR trap* OR catch* OR adhere* OR attach*) (mucous OR mucus OR mucilag* OR exudate* OR resin*) (plant* OR tree* OR sundew Or drosera OR pinguicula OR butterwort OR tarweed) (insect* OR mite* OR athropod* OR spider*)” and using a broader search term: “(adhes* OR stick*) plant”. Thereafter, a search for studies on the use of plant-based adhesives for pest control was done using keywords such as “(plant-derived OR plant-based OR natural OR bio-based) (adhesive* OR glue* OR resin*) (“pest control” OR “pest management” OR “crop protection” OR “biological control”)”. Searches were repeated on May 28^th^ 2025 to include the most recent literature. Other papers were found using Google Scholar and by examining references within papers.

## 2 Evidence for the role of stickiness in plant defense

By now, the role of glandular trichomes and general plant stickiness in direct and indirect defense against abiotic and biotic stress is well recognized (Glas et al., 2012). Some examples: Several genera of the *Nyctaginaceae* family possess glands that produce sticky rings on their stems that form a barrier for ants and other insects (da Cunha Neto et al., 2019). Floral stickiness has been linked to reduced florivory and prevention of nectar robbing (Jaime et al., 2013; Monteiro and Macedo, 2014; McCarren et al., 2021; Chautá et al., 2022). The glandular trichomes of *Madiinae* (tarweeds), *Roridula* spp. and other sticky plants are often covered with carrion (dead arthropods) which may attract predatory arthropods including assassin bugs and spiders. These carrion scavenging predators are often adapted to live on sticky plants. Their presence reduces herbivory and therefore provides indirect defense to the plant (Anderson, 2006; Romero et al., 2008; Riddick and Simmons, 2014; Wheeler and Krimmel, 2015; LoPresti et al., 2018a; Karban et al., 2019; Pearse et al., 2024). Predatory resin bugs even collect sticky plant materials to enhance their own prey capture abilities (Avila-Núñez et al., 2016; Jiménez-Pomárico et al., 2019). The entrapment of sand (psammophory) may also reduce herbivory (LoPresti and Karban, 2016). Likewise, adhesive seed coatings that bind soil particles reduce granivory (Pan et al., 2021). Experimental removal of glandular trichomes or their adhesives generally leads to significant increases in herbivory (Alcalá et al., 2010).

## 3 Morphology and physiology of sticky plants

The book chapter by Adlassnig et al. (2010) provides an extensive summary of the morphology and chemical nature of the traps of carnivorous sticky plants, while Adamec (1997) and Luna-Samano et al. (2024) already discuss the importance of plant nutritional and developmental status. Therefore, the following sections mainly incorporate new insights from studies published after 2010 and information on non-carnivorous plants.

### 3.1 Morphological structure and movement of adhesive secreting organs

In many sticky plants, the glands (or glandular trichomes) that produce adhesive fluids look similar and generally consist of a head containing the glandular cells with a neck cell in the center that is attached to a uni- or multi-cellular stalk (Adlassnig et al., 2010). Glandular trichomes of different lengths may be present on different plant organs and different trichome types may contain different metabolites and fulfill different functions (Adlassnig et al., 2010). In most carnivorous plants with sticky traps, such as *Pinguicula* spp., *Drosera* spp. and *Byblis* spp., the longer glands secrete more viscous fluids with larger adhesive strengths that are primarily involved in prey capture and retention, while the shorter stalkless, sessile glands secrete fewer adhesive fluids that contain larger amounts of digestive enzymes (Huang et al., 2015; Li et al., 2023; Natale et al., 2024).

Movement of the trichomes may also play a significant role in the prey trapping process. For example, in *Drosera*, when prey is captured using the longer trichomes with estimated adhesive strength of 2.197 ± 0.135 N/m^2^ (Huang et al., 2015), the trichomes bend inwards and the leaf curls around the prey to further immobilize them and to bring them in contact with shorter trichomes that secrete the digestive enzymes (**Figure 1C**) (Darwin, 1875). In *Pinguicula* spp., the trichomes are relatively short and cover the leaf surface and flowering stalks (Lustofin et al., 2023). After capturing prey, the trichomes and leaf cells below the prey lose turgor to create a pool of digestive fluids while the leaf edges may also bend inwards (Legendre, 2000). Likewise, the trichomes of *Byblis* spp. vary in length and the longer ones may collapse upon stimulation with prey to perform a similar function (Poppinga et al., 2022). In *Roridula* spp., the dimensions of the glandular trichomes and their physical properties play a major role in the successful capture of arthropods. Out of three general lengths, the longest trichomes are more flexible and less adhesive than the middle-length and short ones, with 17,500, 24,500 and 156,200 median N/m^2^ adhesion strengths respectively. Initial contact with the long trichomes will cause them to bend so that a struggling insect can easily contact the shorter trichomes (Voigt et al., 2009).

### 3.2 Glue chemistry

The type of glue and its physical properties are a major factor in the ability of plants to capture arthropods. The glandular exudates of plants commonly contain a variety of phenols, (mono-, sesqui-, di-, tri-, etc.) terpenes, alkaloids, (poly)saccharides, lipids, fatty-acids, and other compounds (Glas et al., 2012; Muravnik, 2020). Of these compounds, terpenes, essential oils, and sugar esters are well known to have adhesive properties which can help to immobilize arthropods (Wagner, 1991). While the chemical compositions of sticky plant exudates are diverse, there are also similarities between different plant groups.

#### 3.2.1 Acylsugars and terpenes in the trichomes of *Solanaceae*

For plants belonging to the *Solanaceae*, acylsugars and terpenes are thought to play a major role in the stickiness of their trichome exudates. In *Lycopersicon* (tomato) species, different non-glandular and glandular trichome types occur. The type IV trichomes (especially of wild varieties) contain high amounts of acylsugars and smaller amounts of terpenes, while type VI trichomes contain mostly terpenes and methyl-ketones (Tabary et al., 2024a). The sticky exudates of these two types of trichomes function as deterrents and traps for various small arthropods including caterpillars, aphids, whiteflies, spider mites, and thrips (Goffreda et al., 1989; Simmons et al., 2003, 2004; Lucini et al., 2015; Blanco-Sánchez et al., 2021; Narita et al., 2023; Popowski et al., 2024; Tabary et al., 2024a, b). Similar acylsugar-rich trichomes help to protect potato (e.g., *Solanum berthaultii*) plants against various arthropods including aphids, leafhoppers, beetles, and mites (Tingey, 1991). Instead of being continuously sticky, arthropods first need to contact the trichomes of tomato and potato to make the glands rupture and release their contents, which then rapidly oxidize to immobilize the nearby arthropods (Duke, 1994). In tobacco (*Nicotiana* sp.), the longer trichome types secrete a similar, clear, resinous material containing diterpenes (e.g., cembranoids), acylsugars, phenylpropanoids, fatty-acid derivatives and other compounds (Meyberg et al., 1991; Uzelac et al., 2021; Feng et al., 2022; Sharma et al., 2023). Likewise, the trichomes of *Petunia axillaris* (Nadakuduti et al., 2017), *Salpiglossis sinuate* (Moghe et al., 2017), and trichome-rich *Datura wrightii* phenotypes (Hare and Elle, 2002; Hare, 2005; Hare and Smith, 2005; Goldberg et al., 2021) contain high amounts of acylsugars and these plants may be covered with dead insects.

#### 3.2.2 Polysaccharide-rich, water-based glues

For carnivorous plants with sticky trichomes, at least two different types of glues have evolved. Carnivorous plants of the order *Lamiales* (*Pinguicula* and *Byblis*) and *Nephentales* (*Drosera*, *Drosophyllum*, *Triphyophyllum*) produce viscous fluids mostly made up of a watery solution of polysaccharides (Hatcher et al., 2020). In *Byblis* spp. (and presumably also *Pinguicula* spp.) pectin is a main constituent of the mucilage (Giuliani and Maleci Bini, 2008; Li et al., 2023). In *Drosera* spp., the mucilage consists of around 4% polysaccharides, a large volume of water, and some other compounds, including *myo*-inositol and inorganic cations (e.g., Ca^2+^, Mg^2+^, K^+^, Na^+^) (Rost and Schauer, 1977; Loewus and Murthy, 2000; Kokubun, 2017; Vanda et al., 2021). The physical properties of the mucilage and the presence of nanostructures and nanoparticles play a large role in the ability of the fluid to stretch and adhere to arthropods (Erni et al., 2011; Huang et al, 2015; Li et al., 2019, 2020). The viscous pitcher fluids of carnivorous *Nephenthes* spp. and glues and seed coatings of other sticky plants that show similar mechanical properties to the fluid of *Drosera* spp., are likely comparable in terms of chemical composition (Bonhomme et al., 2011; Kang et al., 2021; Kreitschitz et al., 2021), although for many plant species this has not been confirmed yet. Polysaccharides such as pectin also occur in the trichome secretions of other non-carnivorous sticky plant species including *Leonotis leonurus*, *Salvia* spp., *Fagonia* spp. and *Cucurbita pepo* var. *styriaca* (Ascensão et al., 1997; Serrato-Valenti et al., 1997; Corsi and Bottega, 1999; Fahn, 2000; Kolb and Müller, 2004). Lipids and other compounds may also be present alongside polysaccharides, as is the case for *Inula viscosa* (Werker and Fahn, 1981). For *Ibicella lutea* and *Proboscidea louisiana* (*Lamiales*), the oily materials secreted from their sticky trichomes, contain mainly glycosylated fatty acids, glycerides, and dammarane triterpenes (Asai et al., 2010).

#### 3.2.3 Resin-based glues of *Roridula* spp.

The other type of glue, produced by the trichomes of the protocarnivorous plants *Roridula dentata* and *Roridula gorgonias*, is a highly viscous, lipophilic resin. This adhesive substance is mainly composed of acylglycerides and triterpenoids, with small quantities of flavonoids and other unknown triterpenols (Simoneit et al., 2008; Wollenweber, 2007). The glue does not contain enzymes, proteins or saccharides. In *R. dentata* glue extracts, the main triterpenoids are dihydroxyolean-12-ene and dihydroxyurs-12-ene, while in *R. gorgonias*, taxeradiol is the major component (Simoneit et al., 2008). In a way, the visco-elastic secretions from the glandular trichomes of *Roridula* spp. are comparable to pressure sensitive adhesives (Voigt et al., 2009).

#### 3.2.4 More complex adhesive secretions in trees and other plants

The glandular trichomes on the leaves, shoots, buds, and flowers of *Robinia viscosa* var. *hartwigii* trees secrete sticky fluids consisting of a mixture of metabolites including lipids, polysaccharides, flavonoids, proteins and alkaloids (Konarska and Łotocka, 2020). Likewise, young shoots of the legume tree *Schizolobium parahyba* are covered in adhesive secretions (made by epidermal cells) that contain terpenes and lipids (mixtures of essential oils and oleoresins) (Paiva et al., 2022). Other trees such as the silver birch (*Betula pendula*), black poplar (*Populus nigra*), black alder (*Alnus glutinosa*), Scots pine (*Pinus sylvestris*) and horse-chestnut (*Aesculus hippocastanum*) have buds covered with small trichomes that secrete sticky resins made up of complex mixtures which include various amounts of terpenoids, fatty acids, phenylpropanoids and flavonoids (Isidorov et al., 2016). For horse-chestnut, a glandular secretion with adhesive strength of up to 204,300 N/m^2^, depending on temperature and humidity, is present most of the year. This adhesive substance can be thought of as an aliphatic hydrocarbon resin which contains 43.4% triterpenoids, 13% flavonoids and a distinctive 20.1% Aliphatic 3-hydroxyacids with long chains in the range of C14-C22 (Isidorov et al., 2016; Voigt et al., 2020). While occasionally trapping small arthropods, bud secretions like these are mainly for protection of the buds against dehydration and other environmental factors. The composition of horse-chestnut mucilage resembles that of the resin of *Roridula gorgonias* and adhesive seed coating of the radiator plant *Peperomia polystachya*, which are also mixtures of resins based on aliphatic esters and carboxylic acids (Frenzke et al., 2016; Voigt et al., 2020). The resin of tarflower (*Befaria racemosa*), with adhesive strength comparable to commercial flypaper glue (40,000 to 50,000 N/m^2^) (Eisner and Aneshansley, 1983; Voigt et al., 2009), is likely similar. However, detailed chemical studies are still lacking, as is the situation for the composition of the adhesive seed coverage of *Pisonia* tree fruits, the exudates of tarweed (*Madia elegans*), the sticky flowers of *Bejaria resinosa* (Chautá et al., 2022) and *Erica* spp. (likely carbohydrate based) (Vlok and Schutte-Vlok, 2003), and glandular secretions of many other sticky plants.

## 4 Visual cues and volatiles for arthropod attraction or repellence

Arthropods use visual and chemical cues to navigate their environment, locate food sources, and avoid danger (Wenninger et al., 2009). The visual appearance of the leaves, flowers, trichomes, and other parts of sticky plants and the volatile compounds they release therefore can act as powerful signals to influence arthropod behavior (Luna-Samano et al., 2024). Carnivorous plants such as *Drosera* spp. and *Pinguicula* spp. make use of different volatile blends to attract prey towards their adhesive leaves and pollinators towards their flowers (Jürgens et al., 2009; Ojeda et al., 2021; Cuevas et al., 2023). Meanwhile, pitcher plants use flower-like scents and extrafloral nectar to lure insects towards their pitfall traps (Mithöfer, 2022). In contrast, the glandular trichomes and exudates of plants that use stickiness primarily for their own defense commonly contain or release volatiles that repel most arthropods, while being attractive towards some (often specialized) arthropods (van Dam and Hare, 1998; Murungi et al., 2016).

In addition to differences in volatile composition between flowers and traps, the color of green leaves seems more attractive to prey than red leaves and UV patterns of flowers and their spatial separation from traps have been suggested to reduce pollinator capture in sticky plants (El-Sayed et al., 2016; Luna-Samano et al., 2024). Next to this, the visual and volatile cues from carrion present on sticky plants may attract scavenging arthropods (Nelson et al., 2020). Whether the presence of carrion on plant surfaces also deters prey is unclear (Lev-Yadun, 2014). Likewise, it is still unknown if herbivorous arthropods can sense the presence of carrion scavenging predatory arthropods and if herbivores then avoid sticky plants where these predators occur (Dicke and Grostal, 2001). To what extent the shiny appearance of mucilage droplets and the red pigments found in the trichomes, and other parts of plants play a role in defense against herbivores, prey and predator attraction, or as a visual cue that prevents pollinators from becoming trapped is still not entirely clear (Jürgens et al., 2015; Mithöfer, 2022).

## 5 Environmental factors affecting capture success

Temperature variations, wind speed, relative humidity, rainfall, light intensity, and the presence of dust or other particles may all affect the physical and chemical properties of the adhesive traps of sticky plants, and hence their effectiveness, as well as the presence and behavior of arthropods that become trapped in them (Zamora, 1995; Obrycki and Tauber, 1984; Nihoul, 1993). In general, plants with sticky trichomes occur in places where the environmental conditions allow for their adhesives to work, either because these conditions were suitable from the start such as in high humidity or moist areas, or because it was possible to adapt the formulations of the sticky fluids, such as is needed in dry environments. Especially for glues that are watery solutions of polysaccharides or that contain other less rigid substances, certain minimum temperatures and levels of humidity are often required for the traps to work as seen in *Pinguicula* spp. and *Drosera* spp. where sticky droplets may change in viscosity based on temperature, may evaporate under low humidity, or may temporarily be washed away by rain (Rost and Schauer, 1977; Crowder et al., 1990; Legendre, 2000). The traps of *Roridula* spp. are more robust. Their resin changes only slightly under temperature ranges of 20-25°C (viscosity and adhesive strength do change at more extreme temperatures) and remains sticky in dry environments and under water for long time periods, even when plants are dead (Voigt and Gorb, 2010a; Voigt et al., 2015). For many sticky plants, information on how abiotic factors affect the chemical composition, adhesive strength, and longevity of their adhesive exudates under natural and laboratory conditions is still lacking.

## 6 Arthropod species, morphology, and behavior in relation to becoming trapped

### 6.1 Successful capture depends on arthropod and plant identity

In general, healthy plants that are not damaged, plants with larger or more complex trapping structures and stronger adhesives, and the traps of multiple plants combined capture more and larger arthropods (Zamora, 1995; Gibson and Waller, 2009). In addition, the morphology and behavior of herbivores, pollinators, and predatory arthropods in relation to trap morphology and adhesive strength are important factors that determine whether these arthropods become stuck or not in the adhesive secretions of sticky plants. The type of arthropods captured by sticky plants are usually a subset of the arthropods that are available in the environment (Gibson, 1991). For example, *Drosera* spp. and *Pinguicula* spp. capture mainly small insects in their polysaccharide glues including flies, springtails, beetles, thrips and leafhoppers. Larger and stronger insects like ants, moths and small damselflies are only captured occasionally (Zamora, 1999; Ellison and Gotelli, 2009; TV Bierman, personal field observations). In contrast, the resinous secretion from the trichomes of *Roridula* captures various arthropods of considerable size, particularly flying ones (Voigt and Gorb, 2008) and pitcher plants capture a lot of ants and not many flying insects (Zamora, 1999).

### 6.2 Morphological and behavioral adaptations of arthropods to avoid becoming trapped

**Figure 2:**
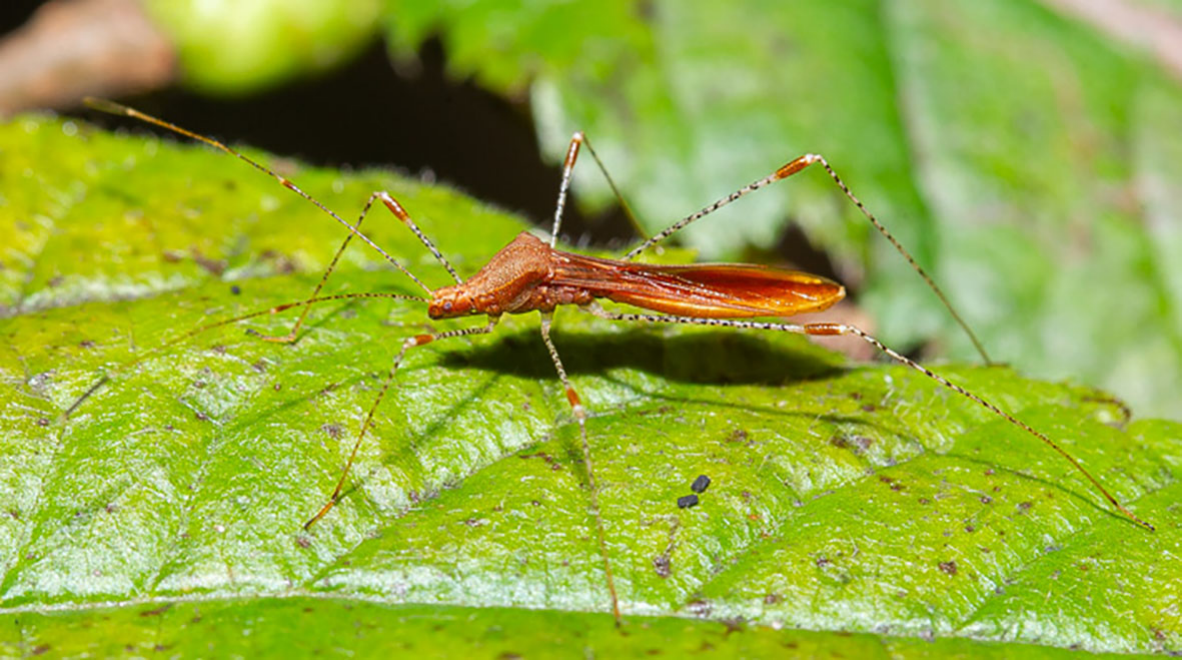
Many Mirid bugs, such as the herbivore *Metatropis rufescens* depicted here, use their long legs to grab onto the stalks of glandular trichomes. In this way, these insects keep their bodies away from the tips of the hooked trichomes and glandular trichomes of their host plant *Circaea lutetiana*, preventing them from becoming trapped. - Photo by Ingmar Van Der Brugge.

Some carrion scavenging arthropods and herbivores avoid being captured and use sticky plants as a habitat and food source. Often this is possible due to behavioral or morphological adaptations. *Pameridae roridulae* assassin bugs (*Miridae*) possess waxy cuticula, strong limbs and a sturdy body which allows them to plow through the adhesives of their host plant *Roridula* (Voigt and Gorb, 2008). Crab spiders living on *Roridula* have also been suggested to use greasy materials to avoid adhering to the trichomes (Marloth, 1903). Likewise, several mealybug species have waxy exteriors which help to prevent them from becoming trapped, for instance while feeding on the sticky flowers and buds of *Erica* sp. (Krimmel and Wheeler, 2015) In contrast, *Dicyphus errans*, another mirid bug that occurs on *Roridula*, avoids contact of its slender body with the trichomes by using its long legs to grab on to trichome stalks and walking over them (Voigt and Gorb, 2010b). Other mirid bugs that live on sticky plants have similar adaptations (**Figure 2**) (Wheeler and Krimmel, 2015; LoPresti and Toll, 2017).

As mentioned before, pollinators may also become trapped in sticky plants. However, temporal, spatial, or chemical separation of traps from flowers and adaptations of pollinators to recognize traps help pollinators to avoid becoming trapped, hence lessening the “pollinator prey conflict’ that sticky plants otherwise face (El-Sayed et al., 2016; Tagawa, 2020). In addition, sticky plants and pollinators likely have evolved adaptations to prevent such conflict from occurring in the first place. For example, some pollinators may have an elongated proboscis to maintain their distance from sticky flowers which may help these pollinators to avoid becoming trapped (McCarren et al., 2021; Chautá et al., 2022).

Other ways in which arthropods can avoid being captured is by detachment of limbs, hairs or scales and by having other protective coatings (Betz and Kölsch, 2004; Krueger et al., 2023). For example, some leafhopper and whitefly species have renewable powder coats that help to prevent adhesion (Gibson and Turner, 1977; Rakitov and Gorb, 2013). Next to this, some arthropods are simply small enough to crawl among the trichomes of sticky plants or may avoid becoming stuck by removing trichomes or their adhesives. Tiny mites and crane fly larvae (*Tipulidae*) move effortlessly along the leaf surface underneath the sticky trichomes of *Pinguicula* plants, while some *Drosera* spp. are home to the trichome devouring larvae of the sundew plume moth (*Buckleria paludum*) and carrion scavenging larvae of the sundew flower fly (*Toxomerus basalis*) (Eisner and Shepherd, 1965; Antor and García, 1995; Zamora and Gómez, 1996; Osaki and Tagawa, 2020; Fleischmann et al., 2022).

## 7 Agricultural applications

### 7.1 Breeding for stickiness

Several crop species and their wild counterparts have sticky features including forage crops like alfalfa, gourds such as pumpkin, hops, cotton, tobacco, tomato, cucumber, potato, cannabis and horticultural household plants like petunia, geranium, and mints (Wheeler and Krimmel, 2015; López-Gallego et al., 2019; Nelson et al., 2019a; Feng et al., 2021). Since glandular trichomes and plant stickiness are involved in several aspects of direct and indirect plant defense against herbivores and environmental factors, breeding to enhance these plant characteristics could be a worthwhile approach to prevent crop losses and improve overall yield (Tingey, 1991; Nelson et al., 2019a). However, many different genes may play a role in trichomes as a defense and engineering trichomes for specific functions may be challenging. Currently, only simple modifications are possible, such as selection for increased content of specific substances via knockout or over-expression of target genes in key pathways. The latter may be achieved using techniques such as CRISPR-Cas9 or modification of plant genomes via *Agrobacterium* (Anilkumar and Chandra, 2025). As research continues, new genomics techniques are developed, and more details regarding the genes and pathways involved in trichome defenses are uncovered, more options should open-up for targeted trichome engineering (Glas et al., 2012; Feng et al., 2021). Of course, one could also consider getting rid of trichomes. Usually this is easier to achieve (e.g., via knock-out of key trichome development genes). In tomato cultivars without trichomes, predatory mites were shown to suppress spider mite and tomato russet mite populations faster and better (Legarrea et al., 2022). This illustrates that with respect to trichome engineering there may be multiple viable strategies to achieve reliable pest control of some target pests in some crop systems.

### 7.2 Sticky plants as a trap crop

**Figure 3:**
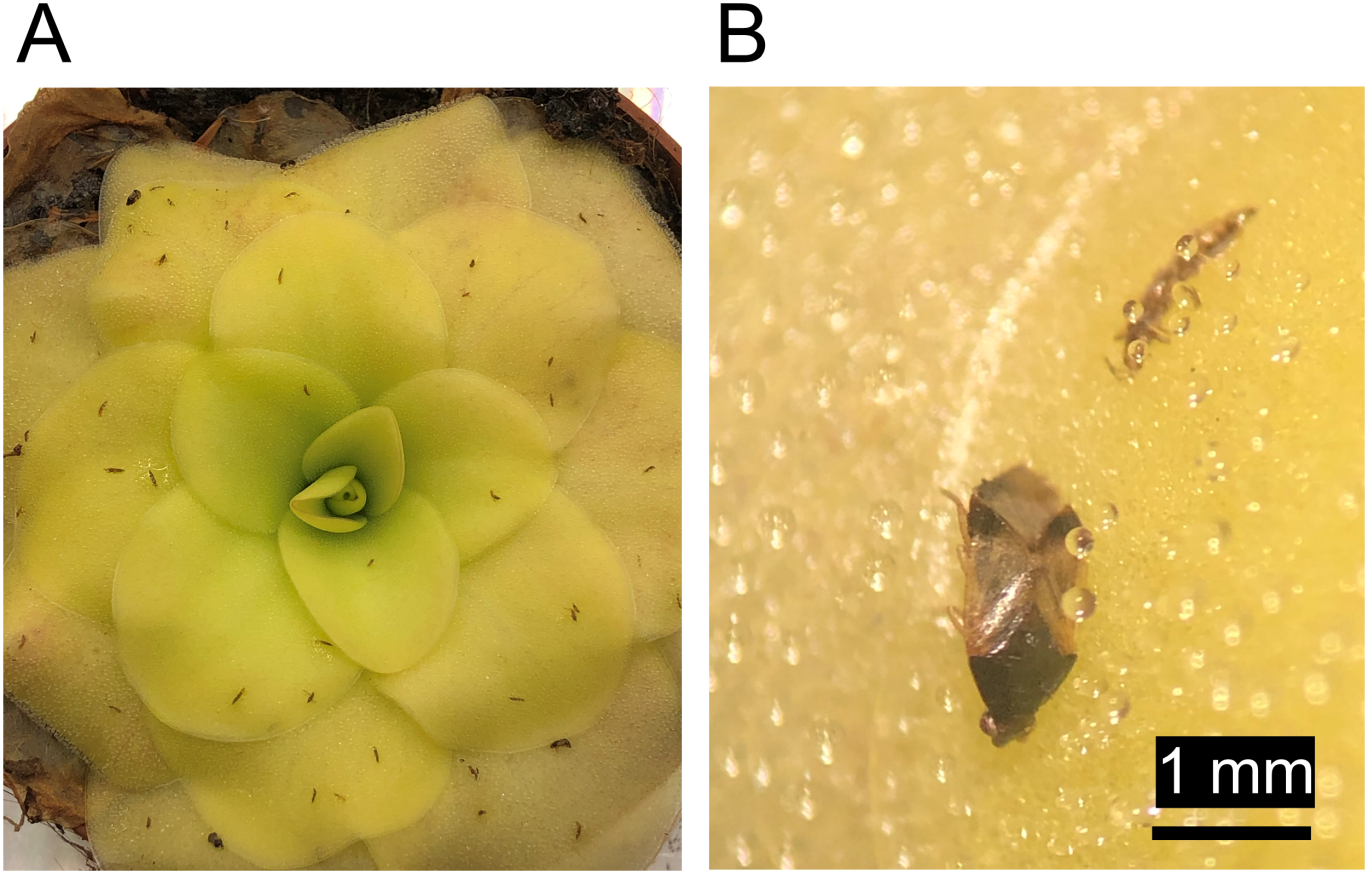
Butterworths (*Pinguicula* spp.) have broad leaves covered with many small trichomes that act as a natural fly paper to trap small arthropods such as flies, springtails, beetles, thrips and leafhoppers. Depicted here is a leaf rosette of *Pinguicula* cultivar ‘Tina’ (*P. agnata* × *P. zecheri*) with trapped western flower thrips (*Frankliniella occidentalis*) and *Orius laevigatus*, a true bug and predator of thrips **(A)** and a close-up of one leaf with a trapped *Orius laevigatus* individual and a trapped western flower thrips individual **(B)**.

Sticky plants passively and selectively trap arthropods that occur in their environment and provide predators with carrion. Small pest insects such as thrips are commonly caught (**Figure 3A**). As such, using sticky crops or sticky companion plants could be useful as a trap crop to protect neighboring plants and for maintaining predator populations in agricultural areas (Sánchez et al., 2003; Tagawa and Watanabe, 2021), although predatory arthropods may also be negatively influenced by stickiness (**Figure 3B**). In one study, a pumpkin cultivar with adhesive trichomes (*Lagenaria siceraria*), was used as a trap crop for whitefly (*Bemisia tabaci*) to protect tomato plants. Mortality rates of whitefly on this pumpkin cultivar were up to 90% and this reduced the population size of whitefly in greenhouse grown tomato by around 45%, from around nine to five adults per leaf (López-Gallego et al., 2019). In another greenhouse trial, a special laboratory strain of tobacco (*Nicotiana benthamiana*) was used as a trap crop and found to be just as efficient as sticky traps in killing whitefly and thrips while having minimal effects on predatory arthropods (Han et al., 2024). Furthermore, this tobacco grew well under several greenhouse conditions and was preferred by whitefly and thrips over other main crops such as tomato and cucumber. Soon after, the resistance of this tobacco strain was linked to acylsugars, creating an opportunity for breeding (Wang et al., 2024). In orchards, *Nicotiana tabacum* may also be useful as a dead-end trap crop for sap-sucking arthropods like the citrus psyllid *Diaphorina citri* which was found to survive less than 30 hours on average on *N. tabacum* (Zheng et al., 2023).

The potential of sticky plants for arthropod trapping is further illustrated by observations made in a teak plantation in India where an undergrowth of malvaceous weeds with sticky trichomes was found to trap and cause high mortality of teak defoliator moth larvae (Loganathan and David, 1999). Natural trapping rates of 25.1-43.9% were observed for defoliator moth larvae of the first three instars. Artificial introductions of first and second instar larvae on plants yielded an even higher rate of 80% mortality already after one day. Likewise, growing sticky legumes in pastures could be beneficial to entrap cattle ticks (Sutherst et al., 1982). Wheeler and Krimmel (2015) furthermore suggested the use of sticky plants in hedgerows to support mirid bugs, other sticky plant specialist predators, and bees and other insects that utilize resin for nestbuilding. The use of carnivorous plants as companion plants for arthropod trapping seems a rather unexplored area. People living in South-Africa hang the branches of *Roridula dentata* and *R. gorgonias* in their house to trap flies (Marloth, 1903), but otherwise carnivorous plants are rarely used for pest control. While for tobacco and malvaceous weeds it seems clear that there is potential, more research is needed before we can conclude whether carnivorous sticky plants can be used as trap crops in agriculture.

### 7.3 Plant-based adhesives as sticky traps

In agricultural settings, sticky traps are an effective method to capture insects like aphids, leafhoppers, and leaf beetles (Straw et al., 2011; Whitfield et al., 2019). The glues used for these sticky traps are still often petroleum based (Hansupalak et al., 2023). However, plant derived materials such as vegetable oils, polysaccharide rich gums or natural rubbers, and resins are already in use and are being increasingly investigated for arthropod trapping. As a result, new advantages of the application of natural glues are being discovered, such as increased adhesive strength and water-resistant properties over conventional adhesives (Maassen et al., 2016; Heinrich, 2019). One of the most well-known plant-based adhesives is tanglefoot. Made of (pine) resin, castor oil, and optional: bees wax and linseed oil, this adhesive has been used since the 1800s for flypaper and is still commonly slathered on tree stems to stop crawling arthropods (Lambert and Franklin, 1967). A recent attempt to use similar glues made from natural rubber, soybean oil and surfactants for sticky traps has resulted in trapping rates of 68% compared to commercial adhesives (Hansupalak et al., 2023). This was mainly due to the glue layer dripping down over time, which illustrates the importance of viscosity in the effectiveness of vertical sticky traps. Another glue made from natural rubber and palm oil has turned out equally or more effective for arthropod trapping than commercially available glues Wiroonpochit et al., 2024). There are no reports yet of sticky traps made with acylsugars, starch, cellulose or other trichome-inspired polysaccharide glues, most likely since these glues have low overall adhesive strengths and are sensitive to humidity and fungi, which are common challenges for polysaccharide-based glues (Heinrich, 2019; Ma et al., 2023).

### 7.4 Plant-based sprayable adhesives for arthropod trapping on plant surfaces

Recently, developments have been made in another trapping approach: sprayable adhesives made from natural materials to provide plants with sticky defenses. With a mode of action similar to the exudates of tomato trichomes, Agri-colle (or Agricolle) is a non-toxic, sprayable glue made from propylene glycol alginate with a physical mode of action. Upon application on infested plants, small arthropods such as aphids, whitefly, psyllids, and spider mites quickly find themselves stuck and suffocating as the substance dries out (Calagri International, 2025) While this approach can be viable to reduce pest numbers by 20% up to around 90% in glasshouse grown crops such as tomato and cucumber (Pilkington et al., 2013), achieving sufficient effectiveness in other systems, such as pear orchards, seems more challenging (Balkhoven, 2009). In addition to Agri-colle, the same producers have developed Agri-50, a formulation based on potassium phosphate and xanthan gum, but this product is not yet available on the market (Calagri International, 2025).

**Figure 4:**
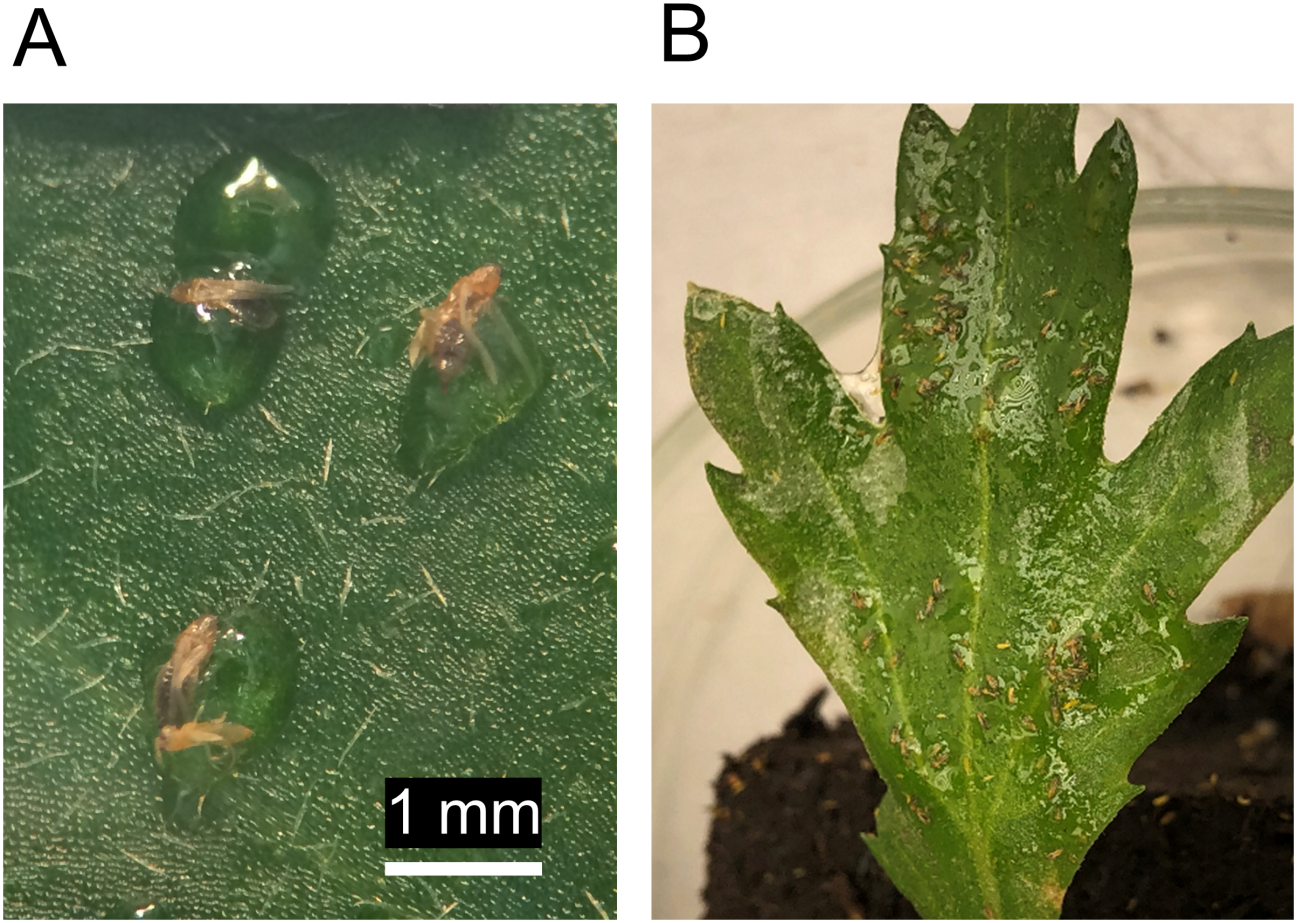
Adhesives made from plant oils are used in various ways in agriculture, including for physical arthropod trapping. Here, western flower thrips (*Frankliniella occidentalis*) female adults are seen trapped in sprayable adhesive droplets made from epoxidized rice germ oil on a chrysanthemum leaf surface **(A)**. A disadvantage of using droplet-based sprays is that uniform coverage is hard to achieve. Using more fluid rice germ oil-based sprayable adhesive formulations, a larger area of the leaf can be covered and large numbers of thrips may be captured **(B)**.

Another development in the realm of bio-inspired sprayable adhesives is the use of alginate-based solutions that contain sticky particles or droplets made from oxidized and crosslinked vegetable oils (e.g., rice oil or mixtures of sunflower-, olive-, and linseed oil) to physically trap small arthropods on plant surfaces. When filter papers and detached leaves were sprayed with these plant oil-based adhesives in laboratory assays, catch rates of western flower thrips (*Frankliniella occidentalis*) of 20% up to 94% were obtained (**Figure 4A**) and thrips damage and reproduction were reduced (Bierman et al., 2024; van Zwieten et al., 2024). In later trials with caged chrysanthemum plants, the trapping effectiveness was lower. However, thrips damage and population growth were still significantly reduced up to 50% on adhesive-sprayed plants (Bierman et al., 2025). These results could be due to direct effects on thrips of the plant oils and the alginate and surfactant of the carrier solution, and due to indirect effects on thrips via plant metabolomic changes that occurred after spraying (Bierman et al., 2025). In continuation of this work, more fluid oil formulations (**Figure 4B**) are currently being tested in combination with predatory arthropods. As continuous efforts are being made to make new bio-inspired adhesives, more eco-friendly pest trapping options will likely soon appear.

A third, very recent development is the use of *Drosera*-inspired natural deep eutectic solvents (NADES) to make polysaccharide-rich sprayable adhesives for control of small arthropod pests (Arunachalam et al., 2025). NADES mixtures of water, glucose, fructose, sucrose and hyaluronic acid were found to trap up to 70% western flower thrips (*Frankliniella occidentalis*) when tested in Petri-dishes in an environment of 60% relative humidity. In addition to their presumed excellent biodegradability, the NADES adhesives seem to have low phytotoxicity on plants and are easy to rinse off plant tissues with water, highlighting the potential of this method for use in greenhouse grown crops (Arunachalam et al., 2025).

### 7.5 Plant-based materials as adjuvants and (nano)carriers of pest repellent chemicals

In addition to their use as a physical trap, plant-based and other natural materials may also be utilized as adjuvants and (nano)carriers to improve the stability, longevity, efficacy and plant surface adherence of botanicals and regular pesticides (Zainab et al., 2024). For example, Iqbal et al. (2022) used a mixture of lemongrass (*Cymbopogon citratus*) oil and *Prosopis juliflora* extracts as an adjuvant to enhance the effectiveness of neem oil application against whitefly on detached eggplant leaves. Likewise, Fang et al. (2023) used *Camellia* oil-based emulsions as nanocarriers to enhance the effectiveness of the pesticide emamectin benzoate against *Spodoptera litura* larvae on detached *Nicotiana tabacum* leaves. Although not necessarily a strong adhesive, the polysaccharide alginate has been used as an adjuvant to reduce pesticide drifts during spraying. When used in the field, this approach led to reduced effects of the used pesticides on pollinators while maintaining efficacy against target pests (Kannan et al., 2024).

Similar sprays made by encapsulation of fluid baits or other active chemicals in alginate hydrogel beads or those made from water-based mixtures containing other (poly)saccharides, proteins or plant oils and sprays featuring hydrogels made of chitin, cyclodextrin, gelatin, carrageenan, pectin, cellulose, or hyaluronic acid can be used to deliver pesticides, botanicals or other control measures (e.g. entomopathogenic fungi and nematodes) more efficiently to various pests, such as mosquitoes, fruit flies, moths and ants, an may also prevent fungal diseases such as mildew (Mason et al., 1998; Böckmann et al., 2014; Tay et al., 2020; Kumar et al., 2021; Sarma et al., 2023; Pavithran et al., 2024; Yayci et al., 2024). Another interesting example is “specialized pheromone and lure application technology” (SPLAT), a cream-like, tacky substance for the controlled release of volatiles. SPLAT formulations, typically made from mixtures of mainly water, wax, and vegetable oils, are successfully used for various agricultural and forestry applications, including mating disruption, mass trapping, attract and kill and repellency of fruit flies and several species of beetles and moths (Teixeira et al., 2010; Mafra-Neto et al., 2013, 2014). In addition to their use as agricultural sprays, natural adhesives may also be used as carriers of pest repellent chemicals to protect stored foods. For example, starch-based adhesives loaded with cinnamon oil were found useful to protect cardboard food packaging against *Plodia interpunctella* larvae (Kim et al., 2020).

## 8 Compatibility of plant stickiness with predatory arthropods and pollinators

An important aspect surrounding the application of sticky plants and plant-based adhesives in crop protection is their compatibility with other IPM practices, including the use of pollinators and biological control agents such as predatory arthropods.

### 8.1 Generalist predators are often hindered by plant stickiness

Predatory arthropods are commonly used in agriculture to suppress pest populations. From an applied perspective, plant stickiness and the application of adhesive substances on plants may have positive and negative effects on predatory arthropods. Plants covered in carrion and pollen due to their stickiness may attract and maintain higher predator populations (Krimmel and Pearse, 2013; Nelson et al., 2019a). In addition, the presence of glandular trichomes may reduce intraguild predation (predators eating each other), a phenomenon that can otherwise considerably reduce the effectiveness of predatory arthropods to suppress pests (Griffin and Yeargan, 2002; Barriault et al., 2019). However, the presence of carrion may also increase cannibalism among predators on some sticky plants (LoPresti et al., 2018a). Some generalist predators also perform better on sticky plants than on plants without trichomes (Krimmel and Pearse, 2014) or may learn to adapt to sticky plants when reared on them (Drukker et al., 1997). However, this seems to be the exception rather than the rule since several studies have shown that the mobility and effectiveness of many commonly used predatory arthropods (e.g., ladybeetles, lacewing and hoverfly larvae, parasitoids, and predatory mites) are significantly hindered by the glandular trichomes of several plant species (Romeis et al., 1999; Gassmann and Hare, 2005; Riddick and Simmons, 2014; Wheeler and Krimmel, 2015; Legarrea et al., 2022; Carretero et al., 2022). The application of adhesive substances on plants will likely come with similar effects although no studies have addressed this so far. In addition, under greenhouse conditions, the negative effects of glandular trichomes and plant-based adhesives on predatory arthropods may be greater than in field situations where environmental factors such as temperature fluctuations, dust, wind, and rainfall may reduce the stickiness of the adhesive trichomes (Obrycki and Tauber, 1984) or of the adhesives.

### 8.2 Sticky plant specialist predators have potential for crop protection

Meanwhile, specialist arthropod predators of sticky plants seem to have less trouble with navigating on plants with glandular trichomes and therefore seem more suitable for use alongside sticky crops (Krimmel and Pearse, 2013) and with plants treated with natural adhesives. In fact, some of these specialist predators are already employed as biocontrol agents or occur freely in agricultural areas, e.g., the stiltbug *Jalysus wickhami* in tobacco (Nelson et al., 2019a, b) and several species of stilt bugs and mirid bugs, including *Tupiocoris cucurbitaceus*, in cultivated tomato (López et al., 2019; Montiel Cáceres et al., 2023). Other predatory arthropods that live on sticky plants remain to be utilized for crop protection, perhaps seeing use after breeding for desired traits (Pérez-Hedo et al., 2021) or may be unsuitable due to these predators also causing feeding damage to crops (Castañé et al., 2011) or eating each other (Moreno-Ripoll et al., 2012; Vila et al., 2012). (For more information on Mirid predators and their potential for agriculture see Wheeler and Krimmel, 2015). Altogether, there seems to be potential for utilizing predatory arthropods, especially specialists, alongside sticky crops and natural adhesives, but management practices may have to be adapted before the use of these predators can be successful on sticky crops.

### 8.3 Non-target effects of plant stickiness and plant-based adhesives on predators and pollinators

Plant glandularity and plant-oil-based adhesives represent a promising and innovative approach to crop protection, offering several environmental, agronomic, and ecological advantages. Commercially used pollinators such as honeybees and bumblebees are unlikely to be detrimentally impacted by plant stickiness as they are of considerable size, have co-evolved with plant stickiness as a defense, and some are used to handle plant resins for their own benefit (Shanahan and Spivak, 2021). Small-sized pollinators such as wild bees, wasps, thrips and minute flies and beetles likely have a higher risk of being trapped while hoverflies, moths and butterflies are less likely to be captured (Zamora, 1999), In contrast, some predatory arthropods will likely face issues with plant stickiness while others will be more compatible.

In addition, despite being of natural origin, the use of natural materials such as plant oils often comes with side effects on arthropods including toxic or sublethal effects (Biondi et al., 2012). When applied, adhesives made from plant oils may therefore potentially interfere with predator survival, development, reproduction, and/or behavior through mechanisms such as the disruption of the nerve system, growth and molting or by having other toxic and repellent properties (Costa et al., 2025), highlighting that the term ‘natural’ does not always equal ‘safe’. In general, however, these effects should be less strong than for chemical pesticides and may also be more specific and stronger towards certain arthropods, possibly allowing for the potential use of plant-based adhesives and predatory arthropods alongside each other in some situations. But there are currently no studies that have investigated such compatibility yet.

## 9 Conclusion and perspectives

In conclusion, stickiness plays a major ecological role in providing plants with direct and indirect defenses as well as nutrition. The successful capture of arthropods in the glandular trichomes of sticky plants is determined by a combination of biotic and abiotic factors. Plant development and nutritional status, the morphology of the adhesive glands (e.g., their surface area, density and three-dimensional structures and mobility), the physiochemical properties of their adhesives, the potential attraction of arthropods through volatile substances and colors, environmental conditions, the specificity of adhesive traps and exudates against arthropods of varying sizes, and the adaptions of arthropods to avoid becoming trapped all are crucial in determining the outcome of the interactions between arthropods and sticky plants. Furthermore, the intricate relationships between sticky plants, the arthropods they trap, and those that associate with sticky plants as herbivores, predators or pollinators adds to the complexity of these natural systems.

Regarding the potential of natural stickiness to enhance the sustainability of current agricultural practices, using sticky plants as trap crops, breeding for enhanced or removed sticky defenses, employment of predatory arthropods that live on sticky plants, and using plant-derived glues for sticky traps, as sprayable adhesives for physical crop protection, and as bio-based carriers for pesticides and botanicals all seem promising options for integration with existing or novel pest management strategies. Based on the case studies presented earlier, using sticky plants such as tobacco as an intercrop or border crop may passively reduce pest pressure via repellence and physical trapping of arthropods while possibly stimulating or having little negative effect on predatory arthropod populations and the environment. Unlike sticky traps, which must be replaced regularly, plants grow, meaning that they provide a continuous source of adhesion and volatiles that are naturally degradable. By breeding for enhanced expression of certain trichome compounds, the innate sticky defenses of crops may be further improved. Likewise, plant-based adhesives may enhance existing pest control methods or provide a new physical trapping mode of action while having less severe effects on non-target organisms compared to conventional pesticides. Integrating plant stickiness further with specialist predatory arthropods may presumably lead to even more effective pest suppression. By harnessing the adhesive properties found in nature and those of bio-based materials, farmers may therefore ultimately very effectively control arthropod pests while minimizing the use of harmful chemicals, thus leading to more sustainable agricultural practices.

However, some major limitations and challenges will first have to be overcome before sticky plants, the trait of plant stickiness itself, the predatory arthropods that occur on sticky plants, and bio-based sticky materials can be applied on a large scale in agriculture. First, the use of sticky plants, their arthropod predators, and plant-based adhesives is currently only partly compatible with current agricultural practices and equipment. In addition, the production of bio-based adhesives and efforts to cultivate and breed sticky crops and their predators are often still small scale. Since the development of new management practices, equipment and facilities for larger scale production take time and money and labor, this may hinder the fast adoption of these new methods by growers and farmers. Second, the use of natural methods does not always produce consistent results, which may lead to reluctance among growers to use such methods. Proper quality control and education may solve these issues, but these usually take time to be implemented. Third, a familiar issue with the use of non-crop plants and bio-based materials is that their production may compete with other societal needs for which space, fertile land and other resources are required. The feasibility for the adoption of stickiness-related methods in agriculture is therefore dependent on whether the use of these methods conflicts with or is compatible with our prior needs as a society and whether consumers also accept these new methods. Finally, we still are lacking knowledge on the side effects of introducing new plant and predator species into our agricultural systems and on how the use of plant-based products affects our crops, non-target arthropods, soil organisms, overall biodiversity, and the rest of our environment. To prevent previous mistakes and future disasters, it is necessary that we inform ourselves well before changing our global agricultural practices.

More research into the ecology, morphology and physical and chemical nature of mucilage producing plants, their secreted adhesives, and how arthropods interact with them may provide us with a better understanding of how sticky plants trap arthropods and may pave the way to future opportunities for the use of sticky plants, their predators, and the development of plant-derived glues for pest control. As more discoveries around sticky plants are being made, new formulations of bio-based glues are created continuously, and our understanding of their unique properties increases, it is likely only a matter of time before plant stickiness and natural glues will be integrated into conventional practices and replace synthetic glues for arthropod trapping.
